# Application of the critical incident technique in refining a realist initial programme theory

**DOI:** 10.1186/s12874-020-01016-9

**Published:** 2020-05-26

**Authors:** U. Cunningham, A. De Brún, E. McAuliffe

**Affiliations:** 1 Mater Misericordiae University Hospital, Eccles St, Dublin 7, Ireland; 2 University College Dublin Centre for Interdisciplinary Research, Education and Innovation in Health Systems (UCD IRIS), School of Nursing, Midwifery and Health Systems, University College Dublin, Dublin, Ireland

**Keywords:** Critical incident interview, Realist, Programme theory, Team, Intervention, Hospital, Methods

## Abstract

**Background:**

As realist methodology is still evolving, there is a paucity of guidance on how to conduct theory driven interviews. Realist researchers can therefore struggle to collect interview data that can make a meaningful contribution to refining their initial programme theory. Collecting data to inform realist Inital Programme Theories (IPTs) in healthcare contexts is further compounded due to the healthcare workers’ busy work schedules.

In this case study of team interventions in acute hospital contexts, we explore the benefits of using the Critical Incident Technique (CIT) in order to build and refine an initial programme theory. We contend that use of the CIT helps to draw on more specific experiences of *“Key Informants”* and therefore elicits richer and more relevant data for realist enquiry.

**Methods:**

The five steps of the CIT were mapped against realist methods guidance and adapted into an interview framework. Specifications to identify an incident as “*c*r*itical”* were agreed*.* Probes were embedded in the interview framework to confirm, refine and/or refute previous theories.

Seventeen participants were interviewed and recordings were transcribed and imported for analysis into NVivo software. Using RAMESES guidelines, Context-Mechanism-Outcomes configurations were extrapolated from a total of 31 incidents.

**Results:**

We found that the CIT facilitated construction of an interview format that allowed participants to reflect on specific experiences of interest. We demonstrate how the CIT strengthened initial programme theory development as it facilitated the reporting of the specifics of team interventions and the contexts and mechanisms characteristic of those experiences. As new data emerged, it was possible to evolve previous theories synthesised from the literature as well as to explore new theories.

**Conclusions:**

Utilising a CIT framework paid dividends in terms of the relevance and usefulness of the data for refining the initial programme theory. Adapting the CIT questioning technique helped to focus the participants on the specifics relating to an incident allowing the interviewers to concentrate on probes to explore theories during the interview process. The CIT interview format therefore achieved its purpose and can be adapted for use within realist methodology.

## Background

As realist evaluation is still evolving, there is a paucity of guidance to support the appropriate use and selection of methods within a realist approach. Whilst researchers are encouraged to use an open, flexible and iterative approach to programme theory development and refinement [1], there is little published on the specifics of *how to* do this. Realist methods are theory driven and in keeping with an iterative and interpretative process. They take into account a broad range of perspectives and seek to deepen understanding of ‘what works, for whom, in what conditions, why, to what extent and how?’ [2]. Realist methods have therefore been increasingly commissioned by health policy makers to inform complex health interventions [3–5].

See Table 1 for a definition of Context, Mechanism, Outcome and Configuration.

**Table 1:** Definition: Context, Mechanism, Outcome Configuration (CMOC)

Context (C)	The conditions in which the programme/intervention is introduced - the enablers/ facilitators/ detractors of team interventions
Mechanism (M)	The process of how the participant interprets and acts upon the intervention stratagem. How any one of the components of a team intervention brings about change. How the resources on offer permeate into the reasoning of team participants.
Outcome (O)	The intended and un-intended consequences of team interventions. Because of the variation in context and mechanisms, there are likely to be different outcomes from teamwork.
Configuration (CMOC)	The patterns and variations in patterns of teamwork*.*

The aim of the realist researcher is to explore theories or hypotheses with regard to *how and why* programmes or interventions work or do not work by engaging in a narrative about the theory with those who have specialist knowledge and are therefore considered *Key Informants*- hereafter referred to as *participants*. This construction of a narrative is usually achieved via an interview process which is designed around the participants’ experience and reasoning with regard to the programme or intervention being evaluated [6]. In order to “theorise the interview”, Pawson advocates for use of Teacher-Learner style interviews where the realist interviewer is urged to take an active role in directing the line of questioning whilst ensuring that the subject matter under evaluation - the programme theory - remains the focus of the interview [7]. For the realist researcher, this can be a demanding task and there is limited guidance in the literature. Manzano’s methods [8] of theory gleaning, refining and consolidation have important application to theory testing in realist *evaluation*, however collecting data for the purpose of building and refining an initial programme theory, is less well explored. In this paper we explore the application of Flanagan’s Critical Incident Technique (CIT) [9] as a technique to elicit the nuance and richness required in developing initial programme theory.

Flanagan describes CIT as “a flexible set of principles that must be modified and adapted to meet the specific situation at hand” [9] p. 335. Specifically,

“CIT research takes place in a natural setting; the researcher is the key instrument of data collection; data are collected as words through interviewing, participant observation, and/or qualitative open-ended questions; data analysis is done inductively; and the focus is on participants’ perspectives” [10] p 16.

The CIT includes five steps in the form of procedures: determination of the general aim of the activity; development of plans and specifications for collecting factual incidents regarding the activity; collection of the data; analysis of the data and interpretation and reporting of the statement of the requirements of the activity. It is apparent from its use in other healthcare studies that these steps can be tailored to specific situations [11, 12]. We sought to explore whether it was possible to maintain this procedural integrity within the more flexible, open and iterative processes required for realist methods.

This technique has already been adapted to explore contextual detail in incidents deemed of critical importance to individuals [13, 14]. As Creswell has already placed CIT in a qualitative framework [15], it has potential to extend its application to realist methods. Woolsey [16] has previously recognised its usefulness in the early stages of research as a foundational exploratory tool and for its role in building theories. We hypothesised therefore that this method could also have potential in this case study. As the CIT allows for in-depth exploration of the antecedents and consequences to a specific incident, it aligns closely to the configuration of context, mechanism and outcomes within the realist evaluation.

Realist methodology calls for interpretive analysis to gain deeper insights on how and why contexts generate outcomes. It is underpinned by a critical realism philosophy [17]. Research in this post-positivist mode requires taking a distanced view [10] and it is accepted that we can identify what we do not directly observe using the practical and theoretical processes of the social sciences.

Chell argues that although emanating from a positivist paradigm, CIT can also be used ‘within an interpretive or phenomenological paradigm’ [18] p 51. Given this, we seek to explore the feasibility and usefulness of quality of data obtained using the CIT. To our knowledge, the application of CIT in realist methods has not previously been examined. This article therefore:

1. Explores use of CIT within a realist methods framework
2. Determines if CIT has potential to be used in refining an initial programme theory
3. Demonstrates application of the CIT via this case study of *team interventions in acute hospital contexts*.

### Contributions to the literature

For the realist researcher, there is a paucity of data on how to conduct interviews for the purpose of refining an initial programme theory.

Although Flanagan’s CIT has been adapted for use in several domains, to our knowledge, its application to realist methods has not been explored.

- Findings contribute to the literature by demonstrating that it is possible to maintain the integrity of the five procedures described in CIT whilst placing the technique into a realist framework. CIT offers researchers a structured, adaptable and practical guide on how realist researchers may apply CIT in refining an initial programme theory.
- The CIT encourages the interviewee to focus on a specific event or incident and in doing so reduces any tendency to provide generic or “woolly” statements that tend to be of limited benefit in developing theory.
- The CIT can enhance initial programme theory development as it facilitates the elicitation of the nuance and specifics of an individual’s experiences and the contexts and mechanisms characteristic of those experiences through the in-depth reflection on those experiences.

Use of CIT allowed participants to recall detail of interventions in an efficient manner. The majority of data retrieved had relevance for initial programme theory building giving a concomitant efficiency to the research team during the data analysis phase.

## Methods

We employed a case study for the purposes of illustrating how the critical incident technique may be applied in realist methods. This study examined the contextual conditions for team interventions in acute hospital contexts and more specifically, the enablers and barriers to team intervention success. It built on previous work which involved a systematic search of the literature using realist synthesis [19].

For the purpose of this research, a multi -disciplinary team intervention was defined as:

*An intervention where a team of two or more disciplines is trying to improve how the team delivers patient care- for example: quality improvement, service improvement or change initiatives; process re-design or team training events*.

These interventions were considered as complex social interventions [2] and realist evaluation was therefore considered an appropriate methodology having already been used in similar studies [12, 20, 21]. The process commenced with a systematic search of the literature which was driven by the researcher’s own experiential knowledge and assumptions [19]. Five plausible hypotheses were extrapolated using realist synthesis and are presented in the form of Context, Mechanism, and Outcome Configurations (CMOCs). Please refer to Table 2 [19].

**Table 2:** Plausible Hypotheses

	**Context**	**Mechanism**	**Outcome**
	If there is:	this enacts:	and results in:
PH1	Inter-disciplinary focus and Flattened hierarchy	Understanding of roles & Mutual respect, support and value Shared decision making and common purpose; self and team efficacy	Increased job satisfaction, higher performance, higher levels of competence, better teamwork and lower feelings of emotional exhaustion. Breaking down of inter-professional silos; more integrated patient care; connectivity of the team and camaraderie.
PH2	Effective Communication: Opportunities for communication; Communication \skills; Communication systems	Shared mental models; Clarity of role; Clarity of purpose	Situational awareness; More integrated care; Better intervention outcomes;
PH3	Leadership Support & Alignment of team goals with organisational goals	Motivates, empowers and engages staff, creating a sense of team efficacy and a shared sense of responsibility and accountability	Team pride; Camaraderie; Connectedness with broader system; Implementation of Intervention; Sustainability of intervention
PH4	Credibility of intervention provided by experienced trainers who team members can relate to and is perceived to be comprehensive (right amount of core topics) with application to the healthcare context in which the team works,	A sense of confidence and engages and motivates team members with the intervention	High satisfaction; Increased skills, Increased self and team efficacy, Increased role in safety and translation to practice.
PH5	Team composition & Physician involvement- consists of appropriately skilled members including a physician, shares a similar foundational knowledge prior to the intervention and participates in a shared learning experience	Shared understanding of the intervention and feel knowledgeable, competent and confident resulting in	Credibility of the intervention, translation to practice and sustainability.

Using these hypotheses as a foundation for the programme theory, during interviews with hospital staff who had been involved in team interventions, the authors sought to explore the conditions in which these interventions were introduced (Contexts- C); how the resources on offer in these particular contexts permeated into the reasoning of those involved in the team intervention (Mechanisms-M) and the intended and un-intended consequences of the intervention (Outcomes- O).

**Fig. 1:**
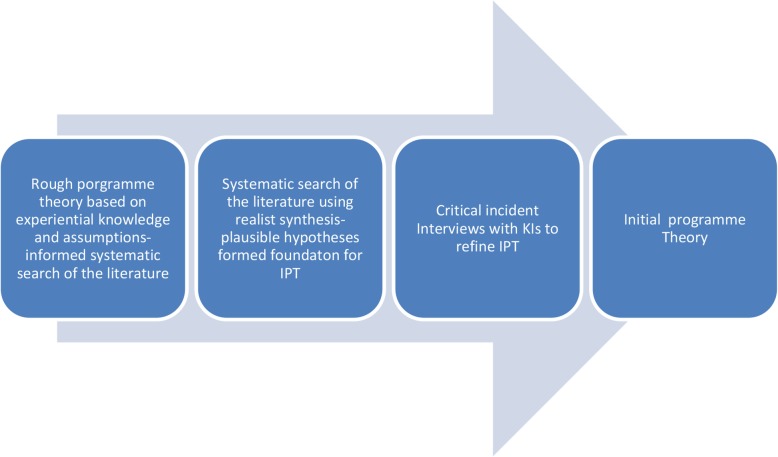
Overview of the initial programme theory development process

Please refer to Fig. 1.

Difficulties for the realist researcher in conducting research have already been cited [20]. In this case study, the busyness of the acute hospital had potential to further impact the fieldwork process. Scheduling interviews during daily routines meant hospital staff had to consciously shift their mind-set from clinical or operational activity to the more reflective mode required for research interviews.

Prior to consideration of the CIT, trial interviews using a semi-structured format had been piloted with two purposively sampled hospital staff -one female hospital operations manager and one female hospital therapist both of whom had led on team interventions. These semi- structured interviews included open ended questions for example:

*“Tell me about an intervention that you have been involved in”* and *“How did the team operate?”*

The research team reviewed the data that emerged from these interviews and agreed that significant portions of the narrative consisted of tangential generalities about teams rather than specific information related to the intervention. Participants found it difficult to construe the intervention, speaking about the team in an abstract and sometimes detached way, as evidenced in one response:

*“I am not even sure of who was involved in that one …some members were only pulled in when they were needed to solve that piece of the puzzle…”* [Semi-structured interview 1]

Participants demonstrated poor recall of specifics about the team or the context in which they were operating focussing instead on the process, problem or issue for which the intervention was designed. In addition, significant tangential information was collected relating to individual work patterns, relationships and practices which had limited relevance for programme theory refinement:

*“Some days are cruel, you know, especially when I am in two different places…I came in this morning at half seven and I haven’t had my lunch yet…I’ve a clinic after this I need to get to, if {Name} is on, I am snookered …”* [Semi Structured Interview 2]

Use of this semi-structured approach had not extracted the necessary detail to meaningfully contribute to programme theory development (i.e., there were insufficient data relevant to context and mechanisms) and the research team agreed that a different format was required.

Researchers agreed that it would be necessary to elicit participant experiences before, during, and after a team intervention/programme was implemented in order to explore contextual conditions for programme theory building [8]. In order to increase the quality and value of the data collected, the researchers therefore considered use of Flanagan’s Critical Incident Technique (CIT) [9] framework.

To ensure rigour in this approach, the research team first mapped and compared the five CIT procedures against the characteristics and features of realist methods (Tables 3 and 4).

**Table 3:** Mapping Critical Incident Technique against Realist Methodology-General principles

**Critical incident technique**	**Adapting for building IPT as part of Realist Evaluation process**
**Incident**- any human observable activity that is sufficiently complete in itself to permit inferences and predictions to be made about the persons performing the act.	**Incident**- team intervention recalled by KI as either a positive or negative experience that meets the criteria in terms of MDT team and intervention descriptors and as specified in the research therefore has potential to contribute to building the IPT
**CIT**- A set of procedures for collecting direct observations of human behaviour- to facilitate their usefulness in solving practical problems and developing broad psychological principles.	**CIT applied**- A set of questions in interview format to elicit factual data to answer my question, what works for whom, in what conditions, why, to what extent and how? Deemed to be useful as it will help identify patterns of regularity in the form of CMOCs
**Critical incidents obtained from interviews** can be relied on to provide a relatively accurate account of job performance.	**Specific accounts of positive and negative experiences** of team interventions obtained from interviews and can be relied on to provide a relatively accurate account of team interventions.
**A set of procedures for analysing and synthesising into a number of relationships that can be tested in more controlled conditions.**	**A set of procedures for analysing and synthesising into a number of chains of inferences (CMOCs) that can be tested**- the IPT will form the basis for further testing
**Obtains a record of specific behaviours**.	**Obtains a record of contexts, mechanisms and outcomes relating to the team intervention.**
**From those in the best position to make the necessary observations and evaluations**	**From Key informants** (participants)- healthcare workers working in multi-disciplinary teams with experience of team interventions in the acute hospital context.
**Incident deemed to be critical**- where the purpose or intent of the act seems to be fairly clear to the observer and where consequences are sufficiently definite to leave little doubt concerning its effects.	**Incident deemed to be critical**-A team intervention recalled by the participant as either a significant positive or negative experience that meets research criteria in terms of multi-disciplinary team and team intervention and therefore has potential to contribute to building the IPT
Recall of factual incidents	Recall of factual incidents
Principal objective of job analysis procedures should be **the determination of critical requirements**, i.e., those that have made the difference between success and failure in carrying out an important part of the job	Principal objective of the analysis procedures in this instance will be **the determination of critical enablers and** barriers for team interventions
**Essentially a procedure for gathering certain important facts concerning behaviour in defined situations**	**Essentially a procedure for gathering certain important facts concerning behaviour in defined situations.**
**Certain more difficult judgments are required** regarding the relevance of various conditions and actions on the observed success in attaining the defined purpose of the activity.	**Certain more difficult judgments will be required** and the research team, the advisory team, and realist support group will be “used” to help in the judgment process of relevance of various conditions on enactment of mechanisms and outcomes
**The incidents must be studied in the light of relevant established principles of human behaviour and of the known facts regarding background factors and conditions operating in the specific situation. From this total picture, the total hypotheses are formulated.**	**The incidents must be studied in the light of relevant established principles of human behaviour and of the known facts regarding background factors and conditions operating in the specific situation.** The interaction of the key informants with the resources on offer by the various contextual conditions impacts their reasoning and mechanisms enacted and subsequent outcomes- **From this total picture, CMOCs are extrapolated and can be interpreted as plausible hypotheses/theories**

**Table 4:** Critical Incident Technique mapped against Realist Methodology for the purpose of the case study

**Establishing the general aim of the activity** Introductory statement explaining the purpose of the study. Request for general aim What would you say is the primary purpose? Request for summary In a few words, how would you summarise the general aim of a specific activity	**Establishing the general aim of the activity** “We are making a study of multi-disciplinary team interventions to improve patient care in acute hospital contexts”. “The primary purpose is to help understand enablers and barriers to success of these interventions” In a few words, how would you summarise the general aim of the team intervention? What were the objectives?
**Purpose and specifications**	**Purpose and Specifications**
Situation Relevance to aim Persons to collect the data need to be familiar with activity.	What was the structure of the team Tell me about what happened and please be as detailed with the facts as possible? Keep in mind the relevance of the team intervention described by the key informant to building programme theory. How data being collected could relate and contribute to programme theory development UC is the primary researcher. Co-author – ADB who is familiar with the research question and purpose of the research was the second interviewer.
**Collecting the data**	**Collecting the data**
Specifications regarding observations	*Specifications as follows:*
• Knowledge concerning the activity • Relation to those observed • Training requirements	• UC trained ADB with regard to the purpose of the critical incident technique and how to unpick the relevance of the team intervention that the key informant is describing to the purpose of the study. ADB is an experienced psychologist and qualitative researcher and is aware of the purpose of the research as co-author. UC drafted the interview and it was reviewed by ADB and EMcA. Both are already familiar with UCs foundational programme theory synthesised from the literature. • UC will complete two trial interviews and send the audio-files for review by ADB and EMcA
**Groups to be observed:**	**Key Informants to be interviewed**
Location Persons Times Conditions	Location Persons Times Conditions
**Behaviours to be observed**	**Detail to be extrapolated** Information relating to contextual conditions and outcomes of the team intervention and *how and why* participants as individuals and as a collective in the team behaved the way they did in these circumstances.
**Rationale for asking for incidents to be recalled as opposed to direct observation**- if suitable precautions are taken, recalled incidents can be relied upon to provide adequate data for a fairly satisfactory for a first approximation to a statement of requirements for the activity. Direct observations are to be preferred but the efficiency, immediacy and minimum demands on co-operating personnel which are achieved by using recalled incident data frequently make their use the more practical procedure.	**Rationale** Hospital workers are extremely busy and the idea of observing in live conditions over the prolonged period of a team intervention will not be practical … a lot of observation could be wasted time as there may be only a couple of critical incidents during a long period of time relating to the research question … this way the participants can be asked for detail of the intervention it relates to the research question and building of programme theory-
**Someone known and respected** by the observer has suggested the interview	**Purposeful sampling by CEO/ General Managers** in 4 hospitals They selected candidates that they think will be able to contribute to the research question i.e., those who had some experience of team interventions either leading or being involved in the team intervention process.
**Questions should be trialled**	**Questions to be trialled.**
Interviewer remarks should be **neutral and permissive** and should demonstrate that he accepts the observer as the expert. Important to get unbiased events	Interviewer remarks should be **neutral and permissive** and should demonstrate that (s)he accepts the observer as the expert. Important to get unbiased incidents
If only giving part of story he should be encouraged by restating the essence of his remarks. This will encourage and help him to bring out many of the details of the incident that the interviewer did not know details of the situation to ask for	During interviews UC and ADB will recall their understanding of what key informants said where necessary requesting clarification or expansion or a response in the form of more detail for example: ***Probe****What was the outcome for patient care in this event… ..The outcome for the team in this event… .. How did you react to this? How did you feel as a result? How did the team react to this? / how did the team feel as a result?*
**Recorded electrically and transcribed**	**Recorded electronically, transcribed and imported into NViVo software**
**Behaviour reports observed by the reporter** Were all relevant factors in the situation given? Has the observer made a definite critical judgment about the relevance of the incident? Has the observer made it clear just why he believes the behaviour was critical?	**Behaviour reports observed by the reporter** Were all relevant factors in the situation given? Has the interviewer made a definite critical judgment about the relevance of the incident? Has the interviewer made it clear how and why he/she believes the contextual conditions generated the outcome and what mechanisms were enacted in doing so.
**Analysing the data and Interpreting and reporting the data.** Imperative reporting is objective.	**Analysing the data and Interpreting and reporting the data.** RAMESES guidelines will be used- inductive, deductive and retroductive logic. Co-authors and realist support group will be consulted to help make judgment calls and challenge thinking.

### Recruitment

Fifteen participants who had been involved in team interventions were purposively sampled initally by either the Chief Operating Officer or General Manager of each participating organisation to reflect a range of disciplines; gender balance and healthcare experience across four acute hospitals in one Irish Hospital Group. Demographic information on participants is presented in Table 5 (Subsequently two additional participants were interviewed to explore an emerging theory further: KI16 & KI17).

**Table 5:** Key Informant Descriptors

**KI code**	**Male/Female**	**Role**	**Healthcare experience in years**
KI1	F	Healthcare Records Document Manager	18
KI2	F	Senior Social Worker Practitioner	12
KI3	M	General Services Manager	2
KI4	F	Clinical Nurse Manager 2	12
KI5	M	Emergency Department Senior Registrar	5
KI6	F	CNM3	25
KI7	F	Administrator and Project lead	20
KI8	F	Operations Manager	25
KI9	F	Physio Operations Manager	14
KI10	M	Consultant & Clinical Director	24
KI11	F	Operations Manager	10
KI12	F	Dietitian Manager	20
KI13	F	Deputy Manager	35
KI14	M	Physiotherapy Manager	21
KI15	F	Staff Nurse	14
KI16	F	Clinical Nurse Specialist, Practice Tutor	14
KI17	M	ED Consultant	26

Participants were invited to participate in the process by e-mail correspondence 1 week in advance of the interviews. The e-mail correspondence included an information sheet and consent form. Participants were advised that participation was voluntary and that their responses would be confidential.

Prior to the interviews being conducted, the researchers considered the application of each of the CIT procedures to the format of the interview.

### CIT procedure 1- development of plans and specifications

The research team clarified the purpose of the critical incident interview technique (as relevant to the case study described) and agreed how to unpick the relevance of the team intervention that the participant was describing. The objective of the CIT interviews was to obtain information relevant to: team descriptors; contextual conditions (C); the objective of the intervention; outcomes (O). Probes were included with regard to how and why an intervention worked in order to elicit the mechanisms enacted (M). The interview guide was designed to give specific attention to the five CMOCs already synthesised from the literature whilst also allowing other contextual enablers and barriers to emerge and the subsequent mechanisms and outcomes generated by those conditions.

### CIT procedure 2-determination of the general aim of the activity

The research team agreed that incidents were “critical” if participants deemed them to be significant in terms of their experience and if they could be relied on as relatively accurate accounts of specific events. A “critical incident” for the purpose of this study was defined as:

“a team intervention **recalled by the participant as either a significant positive or negative experience** that meets research criteria in terms of being a **multi-disciplinary team intervention**”*.*

The team considered that incidents meeting this definition were more likely to meaningfully contribute to building the IPT.

As per the CIT, interviews commenced with an introductory statement to advise participants of the purpose of the exercise (Please refer to Appendix 1. p.1 for complete protocol). Following some background questions regarding professional roles and experience, participants were asked to recall a critical incident as follows:

1. Can you think of a significant event/situation/time that you were particularly proud of working on a team intervention or initiative to improve patient care?
2. In a few words can you tell me what was the primary aim of the initiative was?

If recalled incidents were not deemed to meet the research criteria, participants were re-directed for the purpose of the exercise, for example:

“That’s a really nice example of an intervention introduced with your own professional colleagues, I am going to ask you to think again … .this time if you can think of an intervention where there were a number of disciplines involved, that would be great.”

Following an initial question as to why they selected this experience, they were then asked a series of questions with probes embedded to elicit more factual data specific to the intervention experience. This process was followed by asking participants to recall a significant event /situation/time that they were particularly proud of, and subsequently, one there were not so proud of (*See Appendix**1**Interview)*.

### CIT procedure 3 -collecting the data

#### Tests of critical incident interview format

As with the semi-structured format, the primary researcher (UC) pilot tested the new critical incident interview format on two purposefully chosen hospital staff who had been involved in leading team interventions. Following this, minor changes were made. For example, additional prompts were included to ensure interviewers probed for detail with regard to the existing CMOCs by including prompts for these in a different colour. As per the CIT, it was agreed that interviewer remarks should be *neutral and permissive* [22] and should demonstrate that the interviewee was the expert. However, if specifics were not emerging, clarifications could be sought for example:

“So what you are saying is… .?” or “Can you give me more detail on that?”

Similarly, if information was ambiguous, interviewers could say …

“I am not sure I understood that point, am I correct in saying … .?”

Following the critical incident interview trials, additional probes for data to confirm, refute or refine theories that had worked well were considered and researchers agreed on the final format for the interview (see Appendix 1 supplementary materials for final interview guide).

#### Interviews

Interviews were conducted over a period from May to September, 2018 by two members of the research team (UC & ADB). Interviews were audio-recorded with participants’ consent and transcribed verbatim. One participant did not consent to audio recording and therefore notes were taken by the interviewer during the interview process.

### CIT procedure 4 Analysing the data

Interviews were transcribed, anonymised and imported into NVivo software for the purpose of storage, analysis and interrogation of the data [23].. NVivo memo and annotation functions were used to document thought processes and decision making thus allowing the iterative process of theory building to be captured. This helped to ensure transparency [24–26] thus adhering to RAMESES II reporting standards as well as allowing for the requisite objectivity demanded by the CIT.

A critical incident was the unit of analysis (i.e., each incident described by the participant was one data unit, *N* = 29 incidents, as one participant could not recall a negative experience. Data were subsequently analysed in three phases summarised in Table 6 and detailed thereafter under phase 1, phase 2 and phase 3 headings. Please refer to Table 6.

**Table 6:**
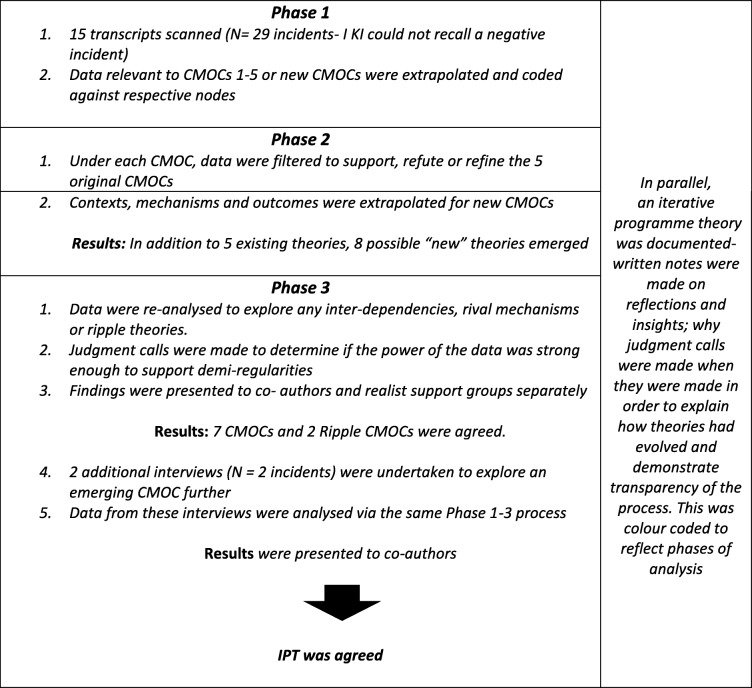
Summary of Data Analysis- Flow chart

As recommended in the Rameses II Quality Standards for Realist Evaluation [27], a retroductive approach was adopted for analysis of the data. Retroduction refers to the movement between inductive and deductive processes to explain *how and why* things work the way they do. Realist researchers seek to explain the hidden causal powers of an intervention in the context in which it is applied [28]. Realist principles of retroduction stress the need for iteration within theory refinement [24]. Going back and forth using both a deductive and an inductive lens enables comparison across units of analysis. The retroductive process used was consistent with the inductive lens required for CIT and also allowed existing CMOCs to be tested using a deductive lens.

Using deductive logic, data were first analysed against the five existing theories (CMOCs) that had been extrapolated from the literature in the form of plausible hypotheses to see whether associations matched the expectations. Consistent with the CIT approach, an inductive lens was also applied to the data as narratives were reviewed to extrapolate evidence that suggested the emergence of new theories. In keeping with the iterative process, the research team and the realist researcher methodology support group* were consulted to provide advice and feedback at each phase of the evaluation process. Use of NVivo software helped to support and document analytical steps and decision-making in line with a retroductive approach to analysis [24].

*Please refer to* Table 7*for overview of consultations sessions.*

**Table 7:** Overview of Consultation sessions

**Sub-group of Co-lead research team** **(Supervisors- Co-authors)**	August 20th- met with Sub-group of the research team i.e., co-authors to discuss Phase 1 analysis
**2nd Author**	August 24th- Consulted with ADB on Phase 1 of coding –agreed nodes in NViVo for analysis: Descriptors, Inductive, Deductive
**Realist Research Support Group**	Sept 12th - Confirmed procedure for data analysis with Realist Support Group
**2nd Author**	Discussed and reached consensus on coding and data extrapolation template.
**Sub-group of Co-lead research team** **(Supervisors- Co-authors)**	Oct 22nd Presented data analysis to date… confirmed methodology going according to plan. Challenged some of conclusions. Suggested further exploration where there was ambiguity over context and mechanisms.
**Realist Support Group**	Nov 6th Presented framework for methodology paper on building IPT and preliminary analysis of data.

* An interdisciplinary group of researchers and academics with a specific interest in, and experience in applying, in realist methods.

#### Phase 1 data analysis: scanning transcripts- induction and deduction

Using the five CMOCs that had emerged from the literature as parent nodes, phase 1 analysis involved an initial scanning of the transcripts. Pieces of narrative were coded according to parent nodes “team descriptors” and “CMOCs 1–5” Nodes were also created to explore new potential CMOCs.

A piece of narrative was annotated if it was judged to be a relevant observation relating to the theory; for example, if it demonstrated a moderating function or appeared to refute, support or confirm prior findings. Where there was evidence of a new contextual enabler or barrier emerging, a memo was written to document how and why it was perceived to be so and to record the rationale for decisions made.

In parallel to the coding process in NVivo, a programme theory template was also developed in hard copy with each phase of the analysis colour coded to demonstrate the evolving theories and/or new emerging theories (*Available on request from primary researcher UC).* This was done because the primary researcher (UC) had a personal preference for reviewing the theories with the research team in hard copy as the evolution of theory refinement was considered easier to view and be interrogated at a glance.

#### Phase 2 data analysis: building and refining theories- Retroduction

Data that were coded under the 5 original CMOCs were re-analysed and re-coded against 3 child nodes: support/ refute/ refine to allow for transparency of the process.

Narratives coded under “New CMOC” in Phase 1 were re-analysed under eight *emerging* theories. *How and why* they resulted in an intended or un-intended outcome was queried. During this process, evidence to support, refute or refine the enabling condition was first extrapolated. For transparency of the process, each of the eight emerging CMOCs were used as parent nodes and narrative was coded if there was evidence specific to Context, Mechanism and Outcome.

#### Phase 3 analysis

As part of the iterative process of data analysis, additional notes were made if there was evidence of moderating influences, rival mechanisms and inter-dependencies. Where refinement of the theory appeared to be indicated, an annotation was made in NVivo as to *how and wh*y the judgment for same was made. Where a judgment call could not be made by the primary researcher, a memo link was created for discussion with co- authors and the realist support group.

Both groups suggested further exploration specific to one possible new theory - “in the moment learning”. Two additional interviews were therefore undertaken in December 2018 with purposively sampled participants who had specific expertise in delivering team interventions using event simulation as a team training intervention. Two additional positive experiences of team interventions (*N* = 2 incidents) were analysed following the same three phase analysis (Total *N* = 31 incidents). Following this iterative process of data analysis, the research team agreed and finalised the initial programme theory.

### CIT procedure 5- interpreting and reporting the data

As per the CIT, it is “imperative” that interpretation and reporting of data “is objective” [22]. For this purpose, RAMESES II for realist evaluation were followed [28]. In addition, the consolidated criteria for reporting qualitative studies (COREQ) was adhered to [29] . In order to understand and agree the underpinning cause of the outcomes observed, data were presented to the research team and the realist support group on two separate occasions so that the chains of inferences (CMOCs) made by the primary researcher could be challenged. This helped to maintain objectivity and rigour in the process of developing insights.

## Results

Findings relating to how the application of CIT complemented realist methods and its potential in refining initial programme theory are presented below. In addition, the benefit of using CIT within this case study is outlined.

### How the application of CIT complemented realist methods

CIT procedures 1 and 2 helped to focus the researchers on the purpose of the interview and that questions must relate to teamwork and building of programme theory (See Appendix 1 p1).

The mapping exercise comparing CIT and realist paid dividends in terms of the quality, relevance and usefulness of the data collected during the critical incident interviews. In contrast to the generalities elicited during semi-structured interviews, participants appeared to have better recall and to connect immediately with the specifics of the team when describing specific interventions:

[KI 2] Pointing to chairs around the room

“The facilities manager would have been involved in it at the time, the healthcare records manager would have been involved in it at the time, I would have been involved in it from a quality and patient safety point of view, the divisional nurse manager was involved …I suppose in this room where we’re sitting now most of the meetings took place”*.*

In addition, drawing on specific interventions appeared to facilitate an emotional connection with the incident. Participants frequently re-constructed scenarios:

[KI 5] “Because we stood up in front of them and presented that we had taken 1600 patients off their waiting lists and of those 1600 patients we had asked them to see I think 112 so we were able to say look of these 1600 patients that were on your waiting lists, … … ... That’s what this programme does for your service… ..and that was the sea change point because the data was so strong and the relationship changed, literally changed almost overnight”*.*

Participants often unconsciously referred to *how and why* things happened the way they did, linking context and outcomes unpacking the mechanisms that were enacted as illustrated below (emphasis and (M) mechanism indication added):

[KI 4] “It is important that they are aware of why we want to do something … and they feel when you engage with them as well that you are appreciating that you know that the importance of their role in the hospital and **it gives them a sense I suppose of value (M)** … . that they are valuable resource but that they are key, they’re key support in the hospital in terms of patient care”*.*

If detail was unclear or did not emerge, interviewers were instructed to seek clarifications and or to elicit detail regarding team behaviours, actions and observations using specific questions:

[*Q9] Has anything changed as a result of this initiative? If so, how? Probe: What was the outcome for patient care in this event… ..The outcome for the team in this event… ? How did you react to this? How did you feel as a result? How did the team react to this? / How did the team feel as a result?*

And these often stimulated considered responses:

[KI 15] “Well I suppose we would have had that level of trust beforehand and it was just a reinforcement of that level of trust (C) … , I think if we hadn’t had that level of trust beforehand and mutual respect beforehand it wouldn’t have happened in [Name of hospital] in the first place (O) … . but it obviously makes you feel more valued in terms of as a peer (M) in terms of your skill levels but that’s something that you build up over time”

In this way, participants were led by the interviewer via open questions as per below.

Q 7 How did the team operate?

Embedded prompts then suggested interviewers might have the opportunity to explore two plausible hypotheses depending on how the participant responded to this question. This helped to ensure the interviewer did not limit the participant’s response.

Q 7 P*robe for PH 1 (Interdisciplinary approach and flattened hierarchy)* and *PH2* (*Effective communication)*

These prompts helped to marry the purpose of the interview which was to refine the initial programme theory *(realist methods)* with participant responses relating to events and facts, i.e., team behaviours and actions associated with the events *(CIT)*.

### Potential of CIT in refining initial programme theory

This technique was successfully used to refine the initial programme theory. Seven patterns of occurrence of CMOCs were elicited from participant narratives. Two consequential CMOCs in the form of “ripple theories” (i.e., where the outcome of one CMOC became a contextual condition to generate a subsequent CMOC) also occurred with regularity. These chains of inference (IPT) are presented in the form of nine “If-then” statements below – See Table 8 below.

**Table 8:** Initial Programme Theory

**CMOC**	**Context**	**+ Mechanism**	**= Outcome**	**Evidence:** **Key Informants**
1 Inter-disciplinary team approach and Flattened hierarchy	*If* Each team member’s voice is heard and considered of equal value	*Then this enacts:* Understanding of roles, mutual respect, support and value Self & team efficacy Perception of shared decision making Common purpose	*resulting in:* Increased job satisfaction Higher levels of competence Better teamwork Lower feelings of emotional exhaustion Breaking down of inter-professional silos More integrated care Connectivity of the team and Camaraderie and More efficient use of time	KIs: 3,4, 5, 6, 9,11, 13,14, 15,17
2 Effective Communication and Shared Understanding of Goals	*If* There is clear, simple, open, honest and timely communication in an appropriate and inclusive environment with SMART goal setting	*Then this enacts:* Shared understanding and clarity of role and purpose; Self- worth and value; Perceptions of confidence and trust in the Intervention	*resulting in:* Positive engagement of the team Situational awareness More integrated planning More efficient use of time and Better chance of success	KIs: 1,3,4,5, 6, 9,10,11,12, 13,14,16,17
3 Leadership support and alignment of team goals with organisational goals	*If* There is genuine leadership support in the form of tangible resources and positive acknowledgement of staff and alignment of team goals with organisational goals through effective engagement and dialogue	*Then this:* Motivates, empowers and engages staff, Enacts a sense of team efficacy; a perception of sense making and a shared sense of responsibility and accountability	*resulting in:* Team pride and camaraderie; Connectedness and confidence in the broader system; Easier implementation and sustainability of the intervention	KIs: 1, 3, 4, 5, 6,7, 9, 10,11,12,13, 14,15
4 Characteristics of intervention that give credibility	*If* The intervention is facilitated/ driven by experienced facilitators who staff can relate to and trust With appropriate clinician involvement where relevant And has perceived relevance to practice with clearly defined goals/outcomes	*Then this enacts:* Team pride and camaraderie; Connectedness and confidence in the broader system; Easier implementation and sustainability of the intervention	*resulting in:* Team pride and camaraderie; Connectedness and confidence in the broader system; Easier implementation and sustainability of the intervention	KIs: 3,4,5,7,,9,11, 12,13,14,16,17
4a Evidence, recognition and celebration of success	*If* there is evidence of a positive outcome and When there is recognition and acknowledgement that an intervention is successful	*Then this:* Empowers motivates and incentivises staff	*resulting in:* Externally perceived credibility in the intervention and subsequent *buy in* With increased likelihood of further engagement and spread of the intervention and/or future team interventions	KIs:3, 8,14
5 Appropriate Team composition and Physician engagement and support	*If* there is broad and purposeful selection of team composition *with* • Physician engagement and support if intervention has a clinical focus	*Then this enacts:* *Feelings of knowledge confidence and competency* *Psychological safety* *and* *Perception of power and influence*	*resulting in:* *Legitimacy of the Intervention* *Better and more timely “buy in”* *Staff satisfaction* *Translation of intervention outcomes to practice* *and better chance of sustainability*	KIs:1,2,3,4,5,6,7,8,9,10,11,12,13,14,15, 16,17
6 Personal Relationships	*If* team members have positive personal relationships or prior experience of a positive working relationship and/or an established social network	*Then this enacts:* Perceptions of Trust Perceptions of Psychological Safety Shared understanding of experiential knowledge of team: ways of working, skill-sets likes and dislikes	*resulting in:* Better engagement in intervention and Easier implementation Ability to progress intervention issues informally Distribution of work according to skill-sets More honest and open communication More integrated planning Quicker recovery from conflicts	KIs:1,2,3,4,5,6, 7,9,11,13,14
7 Inter-professional tensions	*If* there are inter-professional tensions, rivalry and mis -trust	*Then this enacts:* Feelings of frustration; lack of respect; dis-empowerment, perceptions of lack of psychological safety and cynicism	*resulting in:* Failure to progress the intervention, lack of support for the intervention and/or withdrawal from the process	KIs:1,5,6,7,8,10,11,12
7a Escalating mechanisms	*If* There is failure to progress an intervention, lack of support for the intervention and/or withdrawal from the process because of inter-professional tensions	*Then this enacts:* further escalating mechanisms of dis-satisfaction, depletion of energy and resilience and perception of powerlessness	*resulting in:* Greater silo mentality among professions	KIs: 5,7,14

New enabling or disabling conditions were also considered during the first phase of data analysis *(CIT Procedure 4)* and coded as “possible” theories relating to: competing demands; supportive function of the team; *in the moment* learning or source of drive for the intervention. However, researchers did not find a regular pattern of occurrence across the participant narratives to support these chains of inference. During the iterative processes of programme theory development in Phase 2 and 3 (CIT Procedure 4), these contextual variables were either discounted; judged to be intrinsic to another CMOC or judged to be a moderating function to one of the 7 confirmed theories or 2 ripple theories. Participants did not refute any theories synthesised from the literature thus perhaps strengthening them further.

### How CIT benefitted initial programme theory refinement in the study of *team interventions in acute hospital contexts*

New information that had not been synthesised from the literature emerged from participant narratives. By specifically focussing on the antecedents to interventions and seeking the detail on *how and why* they impacted outcomes, *prior working relationships (CMOC 6*) emerged as a key contextual enabler across 6 incidents. By specifically asking for examples of negative experiences *Inter-professional tensions* emerged as a barrier to intervention success across seven incidents *(CMOC 7)*. For some participants, this was conceptualised as specific individuals having the power to de-rail an intervention process *(KI5, KI7, KI11).* On more objective analysis however (CIT procedure 5) and discussion of the detail of narratives with the realist support group, it was agreed that the barrier was more likely to result from the broader issue of inter-professional tensions and rivalries and thus CMOC 7 and CMOC 7a chains of inference were made.

The flexibility of the CIT meant that interviewers could explore existing theories in more detail thus allowing refinement of theory to occur. For example, in Question 5 relating to the structure and function of the team, interviewers were encouraged to probe in relation to *physician engagement PH 5.* From this, a pattern emerged across 6 narratives relating to the mechanism enacted. PH 5 therefore required refinement. For participants it was the enactment of a perception of power and influence by physician engagement that results in legitimacy of the intervention and yielded the better outcome for the intervention.

[KI 4]: Well I mean as I say when you look at the patient pathways to see that there’s senior clinicians involved (C) in the process means that you know that the rest of the team see that it’s taken seriously (M) I think you know what I mean. That it’s given that level of importance and that they know that by having these people engaging they’re listening and they will address the issues for them and working with the patient and for the patient’s journey as well it will improve by having those (O)*.*

Where inter-dependencies between theories seemed to be suggested, the flexibility of the CIT allowed interviewers to explore *how and why* in subsequent interview*s.* For example, use of effective communication and Smart, Measurable, Achievable, Realisitc, Time-bound goals (CMOC 3); and physician engagement and broad team composition *(CMOC 5)* both appear to give a sense of credibility and are thus related to *CMOC 4*. Upon detailed exploration with participants, these conditions appeared to be associated with a better chance of success of the intervention and it is, this **association with success** that causes high self and team satisfaction; increased team skills, increased team efficacy leading to a successful outcome and positive team reputation and this has a further escalating effect:

… empowering motivating and incentivising staff resulting in externally perceived credibility in the intervention and subsequent *buy in* with increased likelihood of further engagement and spread of the intervention and/or future team interventions *Ripple CMOC 4.*

In the absence of the specific detail that CIT elicits, this important information might not have emerged.

## Discussion

Engaging in a social construction of a narrative about team interventions in acute hospital contexts is challenging and the importance of both the interview process and the interviewer as the ‘prime research instrument’ cannot be under-estimated [7, 8]. As opposed to a positivist approach, where the assumption is that “the researcher is independent of and neither affects nor is affected by the subject of the research” [30] p33, during the realist interview process, the interviewer is considered integral in the development of the theory [1, 8]. In the early stages of programme theory development, the realist interviewer needs to be mindful however that they do not lead the *Key Informants* who are considered the experts in the subject matter and striking this balance can be a challenge.

For this case study, busy hospital workers were required to detach themselves from their daily operational routine in order to get into the reflective mode of the interview and this proved difficult. During the semi-structured interviews, the participants required repeated re-direction to the topic by the interviewer (*SSI 1 & SSI2).* Qu [31] demonstrates how this can jostle with the interviewees thoughts:

"the flow of the interviewee’s story can be inadvertently disrupted by the interviewer, such as by redirecting the narrative or interrupting it… " p.248

In comparison, during the critical incident interviews, the interviewer guided participants towards one positive and one negative experience of a team intervention at a time. Rather than general impressions and trying to access their memory of the event, participants focussed on the specifics of that particular incident. In this way, use of CIT allowed the interviewee to focus their attention on telling their experiences of the positive or a negative incident, allowing detail to emerge organically whilst enabling the interviewer to probe for data that would support, refute or refine theories and/ or probe for new theories. This contributed to the quality, richness and usefulness of the data for refining the initial programme theory.

Little has been published on the efficiency of interviewing and the importance of this for healthcare workers in busy acute hospital contexts. Using the CIT, we found that as interviewers we could help participants access their memories of contextual conditions and outcomes in an efficient manner helping memories to re-surface by stimulating a “*deep dive”* on their thoughts as to how and why these things happened. Stimulating recall of specific incidents created an efficiency in the process. The average interview lasted forty-five minutes as opposed to over an hour for semi-structured interviews. In contrast to the semi-structured interviews, the majority of data retrieved had relevance for initial programme theory building, giving a concomitant efficiency when it came to the analysis phase for the researcher.

CIT literature [25] has demonstrated its evolving application “to focus more on thoughts, feelings, and why participants behaved as they did … in order to build on the practice of focusing on what a person did, why he/she did it, the outcome” (p490). In this study, we sought to understand the subjective reality of participants involved in team interventions in acute hospital contexts in order to gain insights into their motives, actions and intentions in a way that is meaningful. The critical incident interview format allowed interviewers to make connections between contexts (antecedents) and outcomes (consequences) of team interventions. Sometimes these emerged organically and on occasions through carefully embedded probes. Causal explanations of how and why team members choose as individuals and as a collective to behave the way they do under different contextual conditions could therefore be inferred. Realist researchers are often challenged trying to find mechanisms to connect contexts and outcomes. From the critical incident interviews, insights into generative causations were reasonably easy to develop. Participants on occasions unconsciously linked the contexts to outcomes and identified the mechanisms enacted themselves in these situations.

CIT therefore aligns well to Pawson’s theory driven Teacher-Learner style interviews [7] and complements Manzano’s [8] interview methods which are more directive and tailored towards theory evaluation or testing. The advantage of CIT has previously been cited [32] as “its capacity to explore differences or turning points; its utility as both a foundational/exploratory tool in the early stages of research and its role in building theories or models” (p480). CIT is particularly “suited to the exploration of dilemmas or looking at two sides of behaviour—good and bad; effective and ineffective; avoidable and unavoidable …” [11] p102. During critical incident interviews, interviewers extracted information from KIs without judgment. By inviting participants to focus on both negative and positive experiences of team interventions, CIT allowed a balanced approach to data collection. Researchers rarely publish detail of failed interventions and this may account for why barriers to team interventions had not previously been extrapolated from the literature. By drawing on negative experiences, a pattern of evidence emerged to support a theory relating to *Inter-professional tensions* and how and why they might impact on the success of team interventions. *(CMOC 7)*.

Realist methods are underpinned by a critical realism philosophy. Manzano suggests that this ontological view will influence epistemology and choices in research design [8]. Although individuals’ reasoning cannot be seen, and has no external reality, these social phenomena have important practical application for team interventions. Understanding the reasons for certain phenomena in the form of an initial programme theory is a precursor to recommending a change in approach. Using CIT in this case study allowed identification of these causal explanations of regularities and irregularities in the social phenomena associated with team interventions. Whilst the interpretative approach of CIT is in keeping with the realist interpretative approach, the application of CIT in realist methods to date had not been explored.

### Strengths

For staff in busy acute hospital contexts, variables pertaining to team interventions for example the composition of the team completing the intervention, team dynamics, team communication or organisational supports are rarely considered. This study demonstrates how use of CIT within realist methods allowed participants to consider these themes by channelling their recall towards specific incidents and the antecedents and consequences of those incidents, thus contributing to building an initial programme theory. The successful application and feasibility of the use of CIT for this purpose and in this setting is an important finding.

Manzano [8] encourages researchers to develop and share knowledge pertaining to the craft of interviewing. The results of this study demonstrate that CIT can effectively be applied within a realist methods framework. Authors have tried to demonstrate how they have respected the procedural integrity of CIT while embracing its inherent flexibility [18]. By sharing our experience of the use of CIT in developing an initial programme theory and developing a guide for the realist researcher, *(*Please refer to Appendix 2*),* we hope this provides some practical advice and guidance for researchers who may face similar challenges.

### Limitations

In this case study, CIT was used to interview a purposively sampled group of hospital workers. The characteristics of this homogenous group from one hospital group in Ireland which had recently engaged in improvement work may have influenced their propensity to recall relevant material. Future research might therefore explore the application of CIT in realist methods in other healthcare contexts or with healthcare staff not involved in improvement work.

## Conclusions

It was possible to adapt Flanagan’s five CIT procedures as a technique to help refine an initial programme theory. In particular, the CIT helped to stimulate clearer memories with regard to antecedents and consequences of specific team interventions and enabled data to be collected in a time efficient manner. CIT offered a balanced method of eliciting data from participants, allowing detail to emerge organically whilst still allowing flexibility for interviewers to probe for data relating to previous theories extrapolated from the literature. The CIT procedure should therefore be considered for use more widely within realist methodology and the authors have developed a practical guide to support other researchers in application of this approach.

## Supplementary information

**Additional file 1.** Appendix 1- Critical Incident interview format. Appendix 2- Application of the Critical Incident Technique- a Guide for the Novice Realist-Researcher.

## Data Availability

All data generated or analysed during this study are included in this published article and/or previous article [19] and its supplementary information files. A file reflecting the iterative programme theory development process is available on request from the primary author at ucunningham@mater.ie.

## Abbreviations

C: Context
CIT: Critical Incident Technique
CMOC: Context Mechanism Outcome Configuration
KI: Key Informant
IPT: Initial Programme Theory
M: Mechanism
O: Outcome
PH: Plausible hypothesis

## References

[1] Pawson Ray (2006). Digging for Nuggets: How ‘Bad’ Research Can Yield ‘Good’ Evidence. International Journal of Social Research Methodology.

[2] Hewitt G, Sims S, Harris R (2014). Using realist synthesis to understand the mechanisms of interprofessional teamwork in health and social care. J Interprof Care.

[3] Patterson SD (2013). Putting on white coats: professional socialization of medical students through narrative pedagogy in standardized patient labs.

[4] Allan HT, Brearley S, Byng R, Christian S, Clayton J, Mackintosh M (2014). People and teams matter in organizational change: professionals’ and managers’ experiences of changing governance and incentives in primary care. Health Serv Res.

[5] Kirst M, Im J, Burns T, Baker GR, Goldhar J, O’Campo P (2017). What works in implementation of integrated care programs for older adults with complex needs? A realist review. Int J Qual Health Care.

[6] Dalkin SM, Greenhalgh J, Jones D, Cunningham B, Lhussier M (2015). What’s in a mechanism? Development of a key concept in realist evaluation. Implement Sci.

[7] Pawson R (1996). Theorizing the interview. Br J Sociol.

[8] Manzano A (2016). The craft of interviewing in realist evaluation. Evaluation..

[9] Flanagan JC, Flanagan JC (1954). The critical incident technique. Psychol Bull.

[10] Whittaker (2000). The political in qualitative methods. Curr Anthropol.

[11] FitzGerald K, Dent B, Kerins CA (2008). The critical incident technique: a useful tool for conducting qualitative research. J Dent Educ.

[12] Butterfoss FD, Kegler MC, Francisco VT (2008). Mobilizing organizations for health promotion: theories of organizational change. Health behavior and health education: theory, research, and practice.

[13] Wodlinger MG (1990). April: a case study in the use of guided reflection. Alta J Educ Res.

[14] Dworkin R. The Theory and Practice of Equality. Sovereign Virtue. 2000. p. 407.

[15] Whittaker E (2000). The political in qualitative methods (Creswell’s qualitative inquiry and research design). Curr Anthropol.

[16] Woolsey LK (1986). The critical incident technique: an innovative qualitative method of research. Can J Couns.

[17] Bhaskar R (1978). A realist theory of science.

[18] Chell E, Pittaway L (1998). A study of entrepreneurship in the restaurant and café industry: exploratory work using the critical incident technique as a methodology. Int J Hosp Manag.

[19] Cunningham U, Ward ME, De Brún A, McAuliffe E (2018). Team interventions in acute hospital contexts: a systematic search of the literature using realist synthesis. BMC Health Serv Res.

[20] Marchal Bruno, van Belle Sara, van Olmen Josefien, Hoerée Tom, Kegels Guy (2012). Is realist evaluation keeping its promise? A review of published empirical studies in the field of health systems research. Evaluation.

[21] Butterfoss FD (2007). Coalitions and partnerships in community health.

[22] Flanagan John C. (1954). The critical incident technique. Psychological Bulletin.

[23] Castleberry A (2014). NVivo 10 [software program]. Version 10. QSR international; 2012. Am J Pharm Educ.

[24] Gilmore B, McAuliffe E, Power J, Vallières F (2019). Data analysis and synthesis within a realist evaluation: toward more transparent methodological approaches. Int J Qual Methods.

[25] Welsh E. Dealing with data: using NVivo in the qualitative data analysis process. Forum Qual Sozialforschung Forum Qual Soc Res. 2002;3(2) [cited 2020 Apr 18]. Available from: http://www.qualitative-research.net/index.php/fqs/article/view/865.

[26] Using NVivo to enhance transparency in evaluation | The NVivo Blog. [cited 2020 Mar 13]. Available from: http://www.qsrinternational.com/nvivo/nvivo-community/the-nvivo-blog/how-researchers-use-nvivo-to-enhance-transparency.

[27] Wong G, Westhorp G, Manzano A, Greenhalgh J, Jagosh J, Greenhalgh T (2016). RAMESES II reporting standards for realist evaluations. BMC Med.

[28] Greenhalgh T, Wong G, Westhorp G, Pawson R (2011). Protocol - realist and meta-narrative evidence synthesis: evolving standards (RAMESES). BMC Med Res Methodol.

[29] Tong A, Sainsbury P, Craig J (2007). Consolidated criteria for reporting qualitative research (COREQ): a 32-item checklist for interviews and focus groups. Int J Qual Health Care.

[30] Remenyi D, Williams B, Money A, Swartz E. Doing research in business and management: an introduction to process and method: Sage; 1998.

[31] Butterfield LD, Borgen WA, Amundson NE, Maglio A-ST (2005). Fifty years of the critical incident technique: 1954-2004 and beyond. Qual Res.

[32] Butterfield LD. A critical incident study of individual clients’ outplacement counselling experiences: University of British Columbia; 2001. [cited 2019 Feb 2]. Available from: https://open.library.ubc.ca/cIRcle/collections/ubctheses/831/items/1.0089975.

