# Supplementary material for: Application of the critical incident technique in refining a realist initial programme theory
Source: BMC Med Res Methodol. 2020 May 26;20:131. doi: 10.1186/s12874-020-01016-9 (PMC7249649; doi:10.1186/s12874-020-01016-9)
Supplement: Supplementary file 1 — Additional file 1. Appendix 1- Critical Incident interview format. Appendix 2- Application of the Critical Incident Technique- a Guide for the Novice Realist-Researcher. [file 12874_2020_1016_MOESM1_ESM.docx]

**Appendix 1- Critical Incident interview format**

**Appendix 2- Application of the Critical Incident Technique- a Guide for the Novice Realist Researcher**

**Appendix 1**

**Instructions for interviewer:**

1. *Focus on events and facts (behaviours, actions, observations).*
2. Questions must relate to teamwork and building programme theory relating to team interventions.  *Focus on what worked for whom, in what conditions, why to what extent and how? It is important to get an account of the interviewee’s experiences and reasoning relevant to their context.*
3. *You may need to ask specific questions to elicit detail relating to:*
   1. *Contexts:*

*PH 1 Interdisciplinary nature of team & hierarchical structure;*

*PH2 Effective communication;*

*PH 3 Leadership support and alignment of team goals with organisational goals;*

*PH 4 Credibility of Intervention;*

*PH 5 Appropriate Team composition and Physician engagement,*

- 1. *Mechanisms- what did contexts enact? How did team respond to the resources on offer by the team intervention*
  2. *Outcomes – what was the outcome of the specific intervention/ critical incident the interviewee is referring to?*

***Please remember to audio record the session***

*Introduce self, explain research briefly, check interviewee has read the information sheet, answer any interviewee questions, ask interviewee to sign consent form and advise that you will e-mail scanned copy to them.*

**Introduction**

1. We are interested in your personal reflections on experiences when you were working in a **multi-disciplinary team**  and the team was trying to improve how **the team** delivers patient care.
2. You were nominated because (*name of person who nominated interviewee*) felt you are well qualified to do this and we want to learn from your experiences and others like you who have been involved in these type of team interventions.
3. The purpose of this research is to identify enablers and barriers for teams in these situations.
4. We are particularly interested in events, situations or times that stand out in your memory, for instance, those that you would consider significant events or had significant outcomes – either positive or negative.
5. The information you give me today will be confidential and your anonymity will be protected. Please be as honest and accurate as you can be when you are describing what happened.

***Background***

1. First, can you tell me about your current role?
2. How long have you worked in a multi-disciplinary healthcare team/ teams?
3. Have you been involved in initiatives to improve how the team delivers care?

**Questions and probes**

***Positive experience***

1. Can you think of a significant event/situation/time that you were particularly proud of working on a team intervention or initiative to improve patient care.
2. In a few words can you tell me what was the primary aim of the initiative was?
3. Why do you recall this as a positive experience?
4. Where did the idea come from*? Probe- Was the idea from within the hospital or out-side.*

*Did the idea come from management or from the team itself on the ground or outside of the hospital? Elicit driver, Probe PH 3 & 4*

1. What was the structure of the team? What other roles/professions were part of the team? *PH1 & PH 5 Probe*
2. Tell me about what happened – and please be as detailed as possible.
3. How did the team operate? *Probe PH 1 & 2*
4. If you were to do it again, would you do anything differently? What would **you** do? Can you give me an example of what you mean? Is there anything **the team** could have done differently? *Probe -Is there anything* ***others in the team*** *could have done differently?*
5. Has anything changed as a result of this initiative. If so, how*? Probe What was the outcome for patient care in this event…..The outcome for the team in this event….. How did you react to this? How did you feel as a result? How did the team react to this? / how did the team feel as a result? Mechanisms & Outcomes*
6. What enabled the positive outcome- probe why/ how? Probe -*Contexts and mechanisms*
7. What have you learned from the event? *Probe What could others learn from your experience? What advice would you give others in the same situation? Probe PH, 1,2,3,4,5*

***Negative experience***

1. Can you think of a significant event/situation/time that you were not particularly proud of working on a team initiative to improve patient care.
2. In a few words can you tell me what was the primary aim of the initiative was?
3. Why do you recall this as a negative experience?
4. Where did the idea come from*? Probe- Was the idea from within the hospital or out-side.*

*Did the idea come from management or from the team itself on the ground or outside of the hospital? Elicit driver, Probe PH 3 & 4*

1. What was the structure of the team? What other roles/professions were part of the team? *PH1 & PH 5 Probe*
2. Tell me about what happened – and please be as detailed as possible.
3. How did the team operate? *Probe re: PH1 & PH 2*
4. If you were to do it again, what could **the team** do differently? *Probe: What could* ***you*** *do differently? Can you give me an example of what you mean? Is there anything* ***others in the team*** *could have done differently?*
5. What do you think were the main reasons for the negative event- Probe why/ how, what do you mean? *Probe -Contexts and mechanisms*
6. What have you learned from the event? *Probe -What could others learn from your experience? What advice would you give others in the same situation? Probe PH, 1,2,3,4,5*

**Appendix 2**

| **Guide for the novice Realist Researcher in using the Critical Incident Technique (CIT) to build an Initial Programme theory *(IPT)***  *Adapted from Flanagan’s Critical Incident technique (1954)* |
| --- |
| **Critical Incident Interview - *What is it?***  A procedure used to gather specific information with regard to contexts, mechanisms and outcomes in situations deemed significant to a key informant/ interviewee. It requires development of a set of questions to elicit data to answer your research question, what works for whom, in what conditions, why, to what extent and how?  The questions will be deemed to be useful if they will help to identify patterns of regularity in the form of Context- Mechanism- Outcome Configurations.  **Critical Incident**  The realist researcher must first define what is deemed *a critical incident* in terms of the research question. This should be agreed with your expert/ advisory panel. This panel should include a mix of individuals with expert knowledge in the subject matter and individuals with knowledge of realist methods.  **Purpose of the interview**  To obtain a record of specific behaviours related to the critical incident from Key informants (KIs) - those with expert knowledge of the subject matter.  **How?**  KIs are asked specifically to recall a situation that stands out in their memory either positive or negative, good or bad etc relevant to the research question. The interaction of the key informants with the resources on offer by the various contextual conditions of these incidents impacts their reasoning and therefore the mechanisms enacted and subsequent outcomes. From this total picture, CMOCs may be extrapolated and can be interpreted as plausible hypotheses/theories.  **Who to interview?**  The researcher should consider purposeful sampling by an individual who is respected by the realist researcher and who is in a position to identify the key informant as an expert in the subject matter.  **Prior to the interview**  You will also need to agree who will interview**.**  Pilot tests of the interview format need to be undertaken with format amended until the realist researcher is satisfied that it will achieve it’s purpose.  Interviewers need to be mindful that their remarks should be neutral and permissive throughout and should demonstrate that they accept the Key Informant as the expert in order to get unbiased incidents.  However, during interviews the interviewer may need to recall their understanding of what KIs have said and where necessary request clarification or expansion or a response in the form of more detail.  Interviews will need to be recorded electronically and transcribed.  An information sheet regarding the interview and a consent form should be sent electronically to the KIs as least one week in advance of the interview allowing the KIs time to withdraw from the research if they wish.  **CIT Procedure 1 Establishing the general aim of the activity**  The interview should commence with an overview of the purpose of the research for example:    “We are making a study of ………..”  “The primary purpose is to help understand ……….”  This should be followed by a request for a summary of event / situation the KI is going to describe so that the interviewer can make a decision of its relevance. e.g.  We are looking for examples of….an event/ situation that stands out in your memory   1. In a few words, how would you summarise the event/ situation. 2. What were the objectives   **CIT Procedure 2 Purpose and Specifications**  If deemed to be a critical incident as per your research criteria, the interview may proceed by seeking more detail as per below. If not, the interviewer should clarify the purpose of the activity again and re-direct the KI to find another example.   - Why do you recall this incident as significant? - Tell me about what happened and please be as detailed with the specifics as possible   How and why probes…  The interviewer needs to keep in mind the relevance of the incident described by the key informant to the programme n being evaluated and in particular how the data being collected could contribute to programme theory development. All interviewers must understand the research question and the aim of the activity which is to build the programme theory and this will require training in the interview format.  **CIT Procedure 3 Collecting the data**  The interviewers must be familiar with the purpose of the critical incident technique and how to unpick the relevance of the incident described by the key informant to the purpose of the study.  Data to be collected from KIs needs to be specified with regard to the following   1. Contextual conditions 2. Objective of programme/ intervention 3. Outcomes 4. Probes re: mechanisms…how and why did individuals behave the way they did in various contexts?   Specific attention must be given in the interview format to the researcher’s existing candidate theories/ theories extrapolated from a literature review. This may be done in the form of agreed probes.  It is also important to allow new theories to emerge organically.  **CIT Procedures 4 & 5 Analysing the data and Interpreting and reporting the data.**  Realist and Meta analyses Standards for Realist Evaluation Studies RAMESES guidelines should be used – these will require you to use inductive, deductive and retroductive logic.  The principal objective of the analysis procedures in this instance is the determination of critical conditions for intended and un-intended outcomes through mechanisms generated by eliciting patterns of occurrence/ demi-regularities from the narratives of Key Informants.  Seek to unpack the conditions leading to the specific behaviours and the reasoning behind these behaviours and resultant outcomes.  This requires you to engage with your advisory and/or expert panel in the judgment process of relevance of various conditions on enactment of mechanisms and outcomes. |
